# *RSPH4A-PCDx*: An Index to Predict Lung Function Decline in Primary Ciliary Dyskinesia

**DOI:** 10.3390/arm93040027

**Published:** 2025-08-02

**Authors:** Gabriel Román-Ríos, Gabriel Rosario-Ortiz, Marcos J. Ramos-Benitez, Ricardo A. Mosquera, Wilfredo De Jesús-Rojas

**Affiliations:** 1 Department of Basic Sciences, Ponce Health Sciences University, Ponce, PR 00716, USA; groman25@stu.psm.edu (G.R.-R.); grosario24@stu.psm.edu (G.R.-O.); mjramos@psm.edu (M.J.R.-B.); 2 Department of Pediatrics, McGovern Medical School, University of Texas Health Science Center at Houston, Houston, TX 77030, USA; ricardo.a.mosquera@uth.tmc.edu

**Keywords:** primary ciliary dyskinesia, *RSPH4A*, pulmonary function, decline index, Puerto Rico

## Abstract

**Highlights:**

**What are the main findings?**

- Puerto Rican patients with *RSPH4A*-associated primary ciliary dyskinesia (PCD) demonstrate a measurable annual lung function decline of approximately −0.75% predicted.

**What is the implication of the main finding?**

- Identifying a quantifiable rate of lung function decline in a genetically homogeneous PCD cohort enables targeted clinical surveillance and personalized management.
- The *PCDx* tool provides clinicians with a predictive framework to optimize timing for intervention and may be adapted to other PCD genotypes through future studies.

**Abstract:**

Primary ciliary dyskinesia (PCD) is a rare, genetically heterogeneous disorder that impairs mucociliary clearance and leads to progressive lung disease. This study aimed to characterize lung function decline in a genetically homogeneous cohort of Puerto Rican patients with *RSPH4A*-associated PCD and to develop a clinical tool to predict lung function decline and support transplant referral decisions. We conducted a retrospective chart review of patients (*n* = 25) with a confirmed *RSPH4A* [c.921+3_6delAAGT] genetic variant, collecting longitudinal spirometry data and applying linear regressions to calculate each patient’s individual FEV_1_ decline. The median FEV_1_ at diagnosis was 55%, with a median annual decline of −0.75% predicted. Adults exhibited significantly lower lung function compared to pediatric patients, while no difference was seen between males and females. Based on this observed decline, we developed the Predicted Capacity Decline Index (*PCDx*), an index that estimates the age and time until a patient reaches the 30% FEV_1_ threshold, the point at which lung transplant referral is typically considered. Our findings underscore the need for early intervention and suggest that genotype-specific tools like the *PCDx* may enhance clinical decision-making in managing progressive lung disease in PCD.

## 1. Introduction

Primary ciliary dyskinesia (PCD) is a genetically heterogeneous ciliopathy characterized by impaired ciliary motility, resulting in defective mucociliary clearance [1,2]. This promotes chronic bacterial colonization and recurrent pulmonary infections, which progressively damage the airways and lead to a decline in pulmonary function over time [3,4]. Lung function in adulthood is strongly influenced by respiratory health in childhood; frequent infections and complications early in life often lead to greater decline later on [5,6,7]. In PCD, repeated respiratory infections from an early age result in cumulative airway damage, parenchymal injury, and structural remodeling [5,7]. Bronchiectasis, a hallmark feature of PCD, is present in approximately 50% of school-aged children and nearly all adults, leading to abnormal lung function from early childhood [4,8,9,10].

To date, variants in over 56 genes have been implicated in PCD, reflecting its genetic heterogeneity [11,12]. Genetic variants in the *RSPH4A* gene, which encodes a component of the radial spoke head of motile cilia, are a well-known cause of PCD [13]. This variant disrupts radial spoke assembly, impairing ciliary function and mucociliary clearance [14]. Studies have indicated that patients with the *RSPH4A* [c.921+3_6delAAGT] variation experience a more severe decline in lung function compared to those with other PCD-associated variants [15]. This suggests that *RSPH4A* genetic variants may confer a more severe respiratory phenotype within the spectrum of PCD [13,15,16].

Globally, PCD affects an estimated 1 in 7554 [11,17], and among Hispanics, the prevalence is believed to be 1 in 16,309 [18]. In Puerto Rico, over 69% of genetically confirmed PCD cases are attributed to the *RSPH4A* [c.921+3_6delAAGT] genetic variant, due to a strong founder effect [3,14,18]. However, early diagnosis of PCD remains a challenge, primarily due to the broad spectrum of clinical manifestations and limited access to specialized diagnostic tools. The median age of diagnosis in the U.S. is 5 years old, but this is believed to be more varied and later in other cohorts [19,20]. Previous studies have shown that late diagnoses are associated with worse baseline forced expiratory volume in 1 s (FEV_1_) values in patients with PCD [20,21].

The progressive decline in lung function observed in individuals with PCD is driven by a complex interplay of early-onset bronchiectasis, chronic respiratory infections, delayed diagnosis, and structural remodeling of the airways and lung parenchyma. This deterioration is further accelerated by central apparatus defects, such as those caused by the *RSPH4A* [c.921+3_6delAAGT] genetic variant. As a result, lung transplantation may be required relatively early in life, with the median age for transplantation among PCD patients reported at 43 years [22]. Currently, clinical guidance for lung transplantation in PCD is based on criteria developed for cystic fibrosis, which recommend annual discussions when FEV_1_ falls below 50% predicted, and active listing when FEV_1_ drops below 30% [23]. This approach presents a significant challenge for PCD care, as no validated tools currently exist to predict the rate of lung function decline in this population. Moreover, despite the clinical relevance of *RSPH4A*-associated PCD, longitudinal data describing pulmonary decline in affected individuals remain scarce.

Given the predominance of the *RSPH4A* [c.921+3_6delAAGT] variation, there is an urgent need to characterize its natural history and clinical trajectory. This study introduces the *RSPH4A–Predicted Capacity Decline Index*, also known as *PCDx*, a new prediction tool developed from longitudinal spirometry data in a genetically homogeneous *RSPH4A* cohort. This index, developed using longitudinal lung function data from patients with the *RSPH4A* [c.921+3_6delAAGT] genetic variant, provides a tool for personalized disease monitoring, risk stratification, and early intervention planning. This tool may ultimately guide clinicians in optimizing long-term respiratory care and surveillance in patients with *RSPH4A*-associated PCD, while providing the framework for broader clinical application across the disease spectrum.

## 2. Materials

### 2.1. Study Design

A retrospective cohort study was conducted using data from patients with genetically confirmed *RSPH4A*-associated PCD enrolled at an accredited PCD center in Puerto Rico, where lung function is evaluated 2–4 times per year during follow-up encounters. Data were extracted from electronic medical records, including pulmonary function test results (FEV_1_) and key demographic and clinical variables, such as sex, age group (pediatric < 21 years; adult ≥ 21 years), and time since diagnosis. Time since diagnosis served as a reference point for longitudinal follow-up intervals. Repeated measures per subject were included to model individual trajectories of lung function over time. Pulmonary function metrics were interpreted based on Global Lung Function Initiative reference values and following European Respiratory Society/American Thoracic Society guidelines [24].

### 2.2. Population

A homogenous cohort of 25 patients (*n* = 25) with a confirmed genetic diagnosis of *RSPH4A*-associated PCD were included. All participants were of Puerto Rican descent and carried the same biallelic *RSPH4A* [c.921+3_6delAAGT] founder genetic variant. No other pathogenic PCD variants were identified. The cohort consisted of 8 males and 17 females, with 11 classified as pediatric and 14 as adult patients. Ages ranged from 8 to 62 years, with a median of 24 years of age at the last follow up. Median age of diagnosis was 20 years of age.

### 2.3. Statistical Analysis

To assess overall lung function decline in patients with *RSPH4A*-associated PCD, two types of simple linear regressions with 95% confidence intervals were performed via GraphPad Prism (10.5.0). First, a cohort-level linear regression was conducted by pooling all available FEV_1_ % predicted values and corresponding time points (months since diagnosis) from all 25 patients. The resulting slope, expressed in percent predicted per year, represented the average decline across the entire cohort and served as the basis for constructing the *PCDx*. Second, to explore potential subgroup differences, separate regressions were performed after stratifying the cohort by biological sex (male vs. female) and age groups (pediatrics vs. adults). In addition, individual linear regressions were performed for each patient to estimate personal FEV_1_ decline rates. These individual slopes were then compared between groups using the Mann–Whitney U test. Time was treated as a continuous variable defined in months since diagnosis, and statistical significance was determined using a two-tailed *p*-value threshold of <0.05. Minitab^®^ 19 (19.2.0.0) was used to perform a multiple linear regression analysis to assess whether age and sex were significant predictors of lung function decline within the cohort.

## 3. Results

### 3.1. Patient Characteristics

The study included 25 Puerto Rican patients (*n* = 25) with a confirmed genetic diagnosis of PCD. The cohort comprised 32% males and 68% females, and 44% pediatrics and 56% adults, ranging in age from 8 to 62 years. Among adults, 21% were male and 79% were female, whereas the pediatric cohort included 45% males and 55% females. The median age of diagnosis was 20 years. All patients carried the *RSPH4A* [c.921+3_6delAAGT] genetic variant. Demographic and clinical characteristics are summarized in Table 1.

### 3.2. Baseline Lung Function

A total of 254 spirometry measurements were available across the 25 patients. The median FEV_1_ at diagnosis (patients first visit) was 55% [44–72%] predicted, with values ranging from 19% to 120%.

**Figure 1:**
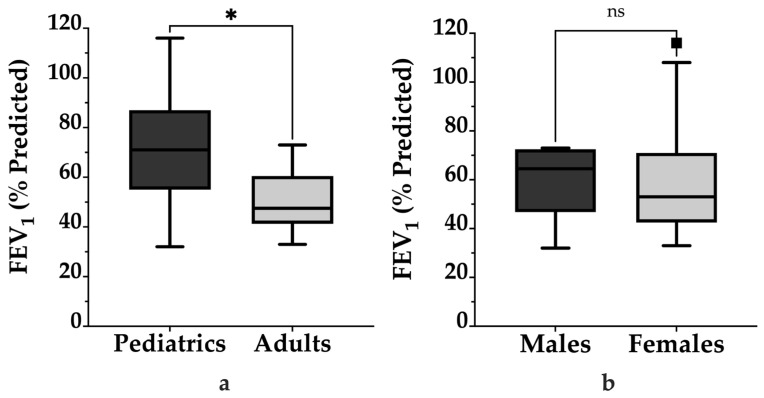
Baseline FEV_1_ percent predicted stratified by age group and sex in patients with PCD. (**a**) Adult patients exhibited significantly lower FEV_1_ values compared to pediatric patients, with a median of 48% [41–61%] predicted versus 71% [55–87%] predicted, respectively (*p* = 0.01). (**b**) Males had a higher median FEV_1_ of 65% [47–73%] predicted compared to females, whose median was 53% [43–71%] predicted (*p* = 0.50). Boxes represent the IQR, horizontal lines indicate medians, and whiskers extend to 1.5 times the IQR. Comparisons were performed using the Mann–Whitney U test. The asterisk (*) indicates statistical significance (*p* < 0.05); “ns” indicates not statistically significant.

Figure 1a shows that when stratified by age group, adult patients exhibited significantly lower lung function at diagnosis, when compared to pediatric patients (*p* = 0.01). Adults had a median FEV_1_ of 48% [41–61%] predicted, while pediatric patients had a median of 71% [55–87%] predicted. Stratification by sex (Figure 1b) revealed that males had a higher median FEV_1_ of 65% [47–73%] predicted compared to females, whose median FEV_1_ was 53% [43–71%] predicted. This difference was not statistically significant (*p* = 0.50).

### 3.3. Longitudinal FEV_1_ Decline

**Figure 2:**
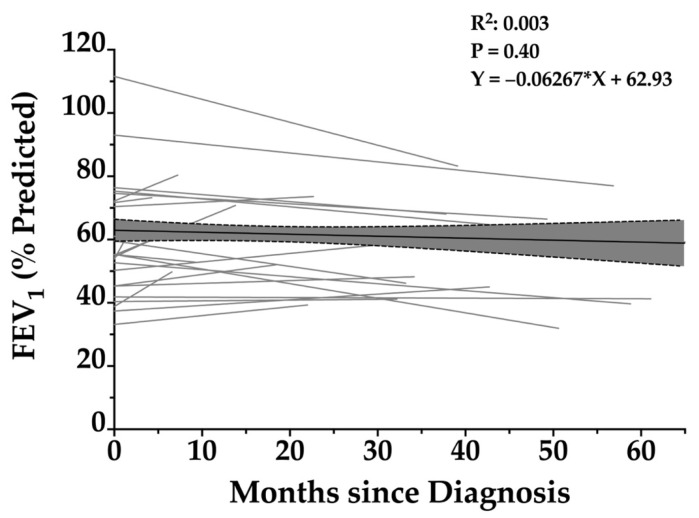
Longitudinal change in FEV_1_ percentage predicted over time in patients with *RSPH4A*-associated primary ciliary dyskinesia. Scatterplot with individual patient trajectories and linear regression lines illustrating the relationship between FEV_1_ (% predicted) and months since diagnosis in patients with PCD. Each gray line represents repeated FEV_1_ measurements over time for a single patient. The black regression line reflects the overall trend, showing a slight, non-significant decline in FEV_1_ over time (slope = −0.06267). The shaded area represents the 95% confidence interval of the regression. The R^2^ value of 0.003 and *p*-value of 0.40 indicate no statistically significant association between FEV_1_ and time since diagnosis.

The cohort-level simple linear regression of all FEV_1_ values plotted against time since diagnosis (Figure 2) yielded a slope of −0.0627% predicted per month, equivalent to −0.75% per year. The 95% confidence interval ranged from −0.2078 to +0.0824, and the result was not statistically significant (*p* = 0.40). The regression equation was as follows:

Y = −0.06267X + 62.93

where X represents months since diagnosis (R^2^ = 0.003). This cohort-level slope served as the foundation for constructing the *RSPH4A–PCDx*.

**Figure 3:**
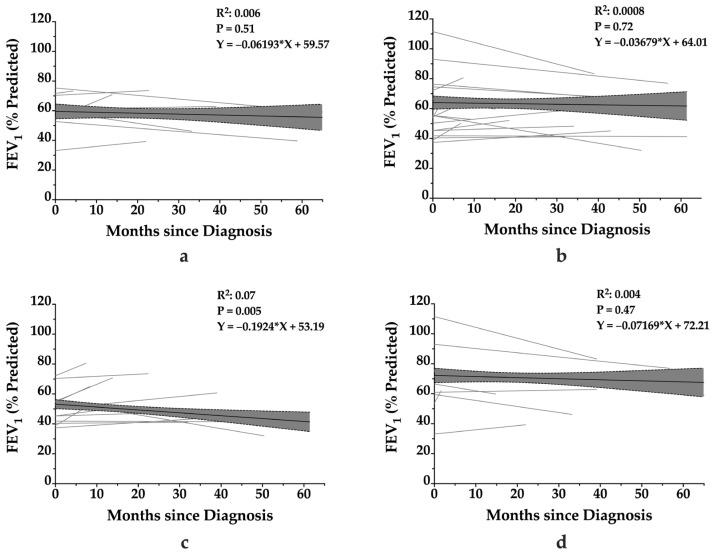
Stratified longitudinal trends in FEV_1_ among patients with *RSPH4A*-associated primary ciliary dyskinesia. Longitudinal changes in FEV_1_ (% predicted) are shown for patients stratified by sex and age group. Panel (**a**) displays data for male patients, (**b**) for female patients, (**c**) for adults (≥21 years), and (**d**) for pediatric patients (<21 years). Each gray line represents individual patient trajectories over time since diagnosis. The bold black line indicates the linear regression trend for each subgroup, with shaded areas denoting the 95% confidence interval. Only the regression model for adults demonstrated a significant association between FEV_1_ and time since diagnosis (*p =* 0.005).

To account for inter-individual variability, individual linear regressions were performed for each patient using their longitudinal spirometry data. The median of these patient-specific FEV_1_ slopes was 0.05% [−0.25–0.36%] predicted per year, with a wide range spanning from −0.73% to 5.04% per year, demonstrating significant heterogeneity in disease progression. When stratified by age group, pediatric patients (*n* = 11) exhibited a median FEV_1_ slope of −0.22% [−0.40–0.28%] per year, while adult patients (*n* = 14) had a median slope of 0.16% [−0.06–1.14%] per year. The difference between the median slope of adults and pediatrics was not statistically significant (*p* = 0.12). Stratification by sex showed that males had a median slope of 0.10% [−0.23–0.30%] per year, whereas females had a median slope of 0.02% [−0.27–0.75%] per year. No statistically significant difference in annual FEV_1_ decline between males and females was reached. Figure 3 shows the linear regressions of all stratifications.

Furthermore, a multivariate analysis consisting of a multiple linear regression was performed to assess the association of age group and gender with annual FEV_1_ slope. Neither gender (*p* = 0.58) nor age group (*p* = 0.98) were significantly associated with FEV_1_ slope, and the model explained only 1.57% of the variance (R^2^ = 0.016).

### 3.4. PCDx

**Figure 4:**
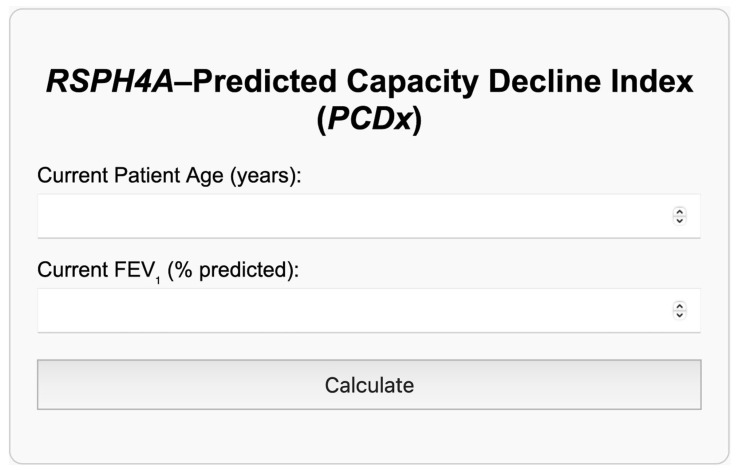
Visual interface of the *RSPH4A*–PCDx. Clinicians input the patients current age and FEV_1_ and using the −0.75% decline described in our study, the index predicts how long and at what age the patient will reach the 30% critical threshold for lung transplantation.

**Figure 5:**
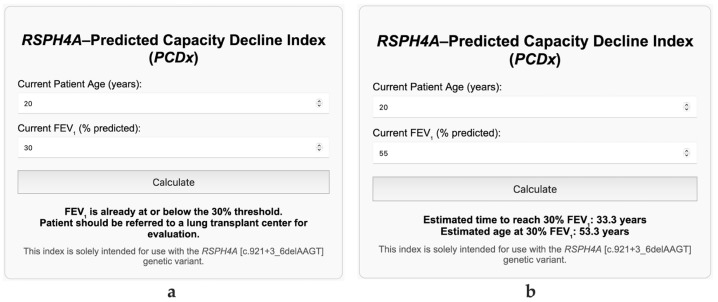
Automated output prompt and disclaimer displayed by the *RSPH4A*–*PCDx*. (**a**) When a patient’s FEV_1_ is at or below the 30% predicted threshold, the index issues a referral prompt recommending that lung transplant evaluation be initiated. (**b**) A fixed disclaimer is displayed indicating that the tool is intended solely for patients with the *RSPH4A* [c.921+3_6delAAGT] genetic variant. Both outputs are automatically generated based on user input and emphasize the clinical applicability and mutation-specific scope of the index.

The *PCDx* was developed as a user-friendly tool to assist clinicians in predicting when patients with the *RSPH4A* genetic variant may reach the critical 30% FEV_1_ threshold for lung transplantation (Figure 4). By inputting the patient’s current age and FEV_1_, the index applies two equations. The first assumes a linear decline in lung function at a fixed annual rate of −0.75%, as observed in our cohort (Figure 2), and is represented in Equation (1). The second equation estimates the patient’s projected age and time to reach the 30% cutoff (Equation (2)). If the inputted FEV_1_ is already below or equal to 30%, the calculator automatically generates a recommendation stating: “FEV_1_ is already at or below the 30% threshold. Patient should be referred to a lung transplant center for evaluation” (Figure 5a). Additionally, a disclaimer is included indicating that this tool is intended solely for use in patients with the *RSPH4A* [c.921+3_6delAAGT] genetic variant (Figure 5b).

Utilizing the median age and FEV_1_ at diagnosis from our cohort, we estimated with the *PCDx* that Puerto Rican patients with *RSPH4A*-associated PCD would, on average, reach transplant-level lung function approximately 33.3 years after diagnosis (Figure 5b).

(1) $$T=\frac{30-FEV1}{r}$$

Equation (1) is the formula utilized by the *PCDx* to estimate yearly lung function decline in *RPSH4A-PCD*. *T* represents the estimated time (in years) to reach 30% predicted FEV_1_, where FEV_1_ is the patient’s current value (% predicted) and *r* is the annual decline rate (−0.75% per year).

(2) Age at Threshold=Current Age+Time

Equation (2) is the formula utilized by the *PCDx* to estimate at which age the patient will reach the 30% FEV_1_ critical threshold. Age at threshold is calculated by adding the patient’s *current age* to *T*, the estimated time to reach 30% FEV_1_.

## 4. Discussion

At the time of diagnosis, the median FEV_1_ in our cohort was 55%, which is notably lower than values reported in other studies, where FEV_1_ medians have ranged from 67.6–71.5% and 44% to 69% predicted [25,26]. However, it is important to note that these previous studies were not limited to genetically homogeneous populations. Our study exclusively focused on patients with the *RSPH4A* [c.921+3_6delAAGT] genetic variant, which affects the central pair apparatus of motile cilia [27]. This genetic variant has been associated with more severe lung disease compared to genetic variants involving the outer dynein arms, such as *DNAH5* [28,29]. Previous genotype–phenotype analyses have shown that patients with central apparatus or microtubular defects experience greater annual declines in lung function than those with outer or combined dynein arm defects [21,30]. Specifically, individuals with biallelic mutations in *CCDC39* and *CCDC40*, genes associated with inner dynein arm and microtubular disorganization, exhibit significantly greater functional impairment over time compared to those with mutations in *DNAH9*, *DNAH5*, or *DNAH11* [31,32].

Our observed annual decline of −0.75% predicted in FEV_1_ mirrors this previously reported trend and further supports the assertion that central apparatus defects confer a worse pulmonary prognosis. Moreover, our cohort’s median age of diagnosis was 20 years—significantly later than the diagnostic age reported in other countries, where the median is often under 5–9 years [17,19,33]. Delayed diagnosis is well recognized as a factor associated with more advanced disease at presentation [21,34]. This may partially explain the poor baseline lung function observed in our cohort and the difference between the pediatric and adult subgroups.

Interestingly, while the cohort exhibited an overall downward trend, some patients showed stability or even improvement in their FEV_1_ after diagnosis. This heterogeneity may be attributed to the initiation of treatment, such as the prompt implementation of airway clearance therapies and appropriate antimicrobial management as described in PCD care guidelines [35]. Additionally, variable environmental exposures, lifestyle factors, and differences in the age of diagnosis may influence individual respiratory outcomes.

In terms of sex differences, our analysis did not reveal any significant differences in lung function decline between males and females. Although females exhibited a lower median FEV_1_ compared to males, this difference was not statistically significant, aligning with findings reported in previous studies [5,10]. However, our study only included a small number of male participants (8 males vs. 17 females), which limits the statistical power to detect sex-based trends and may introduce sampling bias. Regarding longitudinal lung function decline, previous studies have reported annual FEV_1_ declines of 0.18–0.89% predicted per year in PCD patients [5,21,36]. Our finding of a −0.75% predicted yearly decline is consistent with these values and supports the growing body of literature indicating a progressive reduction in pulmonary function in PCD, particularly in individuals with central microtubular defects.

These findings support the development and clinical utility of the *PCDx*, specifically tailored to patients with the *RSPH4A* [c.921+3_6delAAGT] genetic variant. Given the observed annual FEV_1_ decline of −0.75% predicted—the *PCDx* provides a personalized and genetic variant-specific approach to forecasting lung function deterioration over time. By estimating when a patient may approach the critical threshold of FEV_1_ < 30%, commonly considered for lung transplant referral, the tool empowers clinicians to initiate timely interventions, intensify management strategies, and engage in early transplant planning. Furthermore, it facilitates shared decision-making by giving patients a clearer understanding of their disease trajectory. In contrast to other monitoring tools used in chronic lung diseases, such as the Lung Clearance Index (LCI), the *PCDx* was specifically developed to predict the trajectory of lung function decline over time, particularly the expected time to reach critical FEV_1_ thresholds used in lung transplant evaluation. While indices like DOSE (Dyspnea, Obstruction, Smoking, Exacerbation) and BODE (Body mass index, Obstruction, Dyspnea, Exercise capacity) are widely used in COPD to assess mortality risk and current disease severity, they do not model long-term lung function trajectories or guide timing for therapeutic escalation. The *PCDx* fills this gap by providing a simple, spirometry-based forecasting tool, currently tailored to a genetically homogeneous cohort with *RSPH4A*-associated PCD. To our knowledge, this represents one of the only predictive models for lung function decline in a specific PCD genotype. Although a latent class growth model (LCGM) has been proposed to predict decline in COPD patients [37], PCD and COPD differ substantially in etiology, pathophysiology, and clinical course, limiting the generalizability of such models. Therefore, the *PCDx* offers a novel, disease- and genotype-specific approach that may enhance long-term clinical management in PCD.

However, while the *PCDx* provides a genetically tailored tool for forecasting lung function decline, its predictive accuracy may be further enhanced in future research. Incorporating additional clinical variables, such as frequency of exacerbations, adherence to airway clearance regimens, microbiological colonization, and environmental exposures, may improve individual-level prediction. Moreover, the use of machine learning algorithms trained on larger, multicenter datasets could enable more sophisticated, personalized predictive models that account for non-linear trends and heterogeneous clinical trajectories. These advanced approaches hold promise for refining risk stratification and guiding more targeted interventions in patients with PCD. Yet, unlike general predictive models, the strength of the current *PCDx* lies in its foundation of data from a genetically homogeneous population, enhancing its precision and applicability for individuals with the *RSPH4A* genetic variant.

Despite these strengths, this study has several limitations that should be considered when interpreting the findings. First, the retrospective nature of the analysis introduces inherent limitations related to data completeness, consistency of follow-up intervals, and reliance on previously recorded spirometry values. Second, the cohort size was relatively small (*n* = 25) and consisted mostly of female patients, which limits the statistical power to detect subtle differences in lung function trends, particularly in subgroup analyses. Additionally, although meaningful differences in lung function across narrower age categories may exist, our sample size did not allow for reliable stratification beyond the pediatric (<21 years old) and adult (≥21 years old) groups used. Third, because all patients in this study were of Puerto Rican descent and carried the *RSPH4A* [c.921+3_6delAAGT] founder mutation, the generalizability of the *PCDx* to genetically and ethnically diverse PCD populations may be limited.

Fourth, while individual linear regressions provide a practical means to estimate annual FEV_1_ change, this approach assumes linearity over time and does not account for the non-linear progression often observed in chronic lung diseases. More complex modeling strategies, such as mixed-effects models, could offer more precise estimates in future prospective studies with larger datasets. However, given our relatively small cohort (*n* = 25) and exploratory objectives, simple linear regressions were deemed the most appropriate analytical approach for this study. The regression model assessing overall decline (FEV_1_% vs. time since diagnosis) yielded a low R^2^ value (0.003) and was not statistically significant (*p* = 0.40), which limits the empirical strength of the *PCDx*. That said, the absence of statistical significance does not necessarily preclude clinical relevance, particularly in rare diseases where small sample sizes and wide inter-patient variability are common. The observed trend of a −0.75% predicted annual decline remains consistent with previous reports in genetically defined subgroups and underscores the need for proactive monitoring in this population.

Fifth, this study did not account for key clinical variables such as the presence of active infections, acute exacerbations, or adherence to treatment regimens. While all patients were managed according to established PCD care guidelines [35], the *PCDx* operates under the assumption of treatment compliance. Lastly, the *PCDx* developed in this study is based on a single-center cohort and has not yet been externally validated. As such, its predictive utility should be interpreted with caution and only applied to patients with the *RSPH4A* [c.921+3_6delAAGT] genetic variant. Future prospective studies with multicenter collaboration will be critical to validating and expanding the *PCDx* framework. As additional longitudinal datasets become available, incorporating a broader range of clinical variables and applying more complex modeling strategies may further enhance the tool’s accuracy and generalizability across the diverse PCD population.

## 5. Conclusions

This study provides the first retrospective analysis of lung function decline in Puerto Rican patients with PCD carrying the *RSPH4A* [c.921+3_6delAAGT] genetic variant. Our results demonstrate a significantly reduced baseline FEV_1_ at diagnosis and an annual decline of −0.75% predicted, consistent with the progressive impairment observed in patients with central microtubular defects. These findings underscore the severity of lung disease associated with this specific genotype and highlight the impact of delayed diagnosis on long-term respiratory outcomes. This work led to the development of the *PCDx*—a genetic variant-specific tool designed to predict the progression of lung function and anticipate when patients may reach the critical 30% FEV_1_ threshold used in lung transplant evaluation. The *PCDx* offers a practical, personalized approach that supports earlier intervention, improved clinical decision-making, and shared planning between pulmonologists and patients. While the current version of the *PCDx* is tailored to the *RSPH4A* genetic variant, the underlying framework is adaptable and could be applied to other PCD genotypes pending further longitudinal studies. Future studies involving larger cohorts and prospective validation will be essential to refine the *PCDx* and assess its accuracy before it can be fully implemented in routine care. Nevertheless, as additional genotype–phenotype correlations emerge, this model could evolve into a broader predictive platform, advancing the field of precision medicine in PCD and helping physicians tailor treatment plans, guide timely therapeutic escalation, and identify candidates who may benefit from early referral for lung transplant.

## Figures and Tables

**Table 1:** Demographic and clinical characteristics of the study cohort.

Characteristic	Value
Age at diagnosis (years)	20 [13–39.5]
Months since diagnosis	33.5 [11.7–46.2]
*RSPH4A* [c.921+3_6delAAGT]	25 (100%)
Bronchiectasis	25 (100%)
Pediatrics	11 (44%)
Adults	14 (56%)
Male	8 (32%)
Female	17 (68%)

## Data Availability

All data are available upon request through the corresponding author.

## Abbreviations

The following abbreviations are used in this manuscript:

FEV_1_	Forced Expiratory Volume in 1 Second
PCD	Primary Ciliary Dyskinesia
*PCDx*	Predicted Capacity Decline Index
