# Correction: Román-Ríos et al. *RSPH4A-PCDx*: An Index to Predict Lung Function Decline in Primary Ciliary Dyskinesia. *Adv. Respir. Med.* 2025, *93*, 27

**DOI:** 10.3390/arm93050045

**Published:** 2025-10-15

**Authors:** Gabriel Román-Ríos, Gabriel Rosario-Ortiz, Marcos J. Ramos-Benitez, Ricardo A. Mosquera, Wilfredo De Jesús-Rojas

**Affiliations:** 1 Department of Basic Sciences, Ponce Health Sciences University, Ponce, PR 00716, USA; groman25@stu.psm.edu (G.R.-R.); grosario24@stu.psm.edu (G.R.-O.); mjramos@psm.edu (M.J.R.-B.); 2 Department of Pediatrics, McGovern Medical School, University of Texas Health Science Center at Houston, Houston, TX 77030, USA; ricardo.a.mosquera@uth.tmc.edu

Figure Legend

In the original publication [1], there was a mistake in the legend of Figure 1. The legend states that the *p*-value for the difference between adults and pediatrics (second sentence) is *p* = 0.001, but this should be *p* = 0.01. In this same figure, the *p*-value for the difference between males and females (third sentence) is stated as *p* = 0.0078, but this should be *p* = 0.50, as stated in the second paragraph of Section 3.2. Baseline Lung Function. The correct legend appears below.

In Figure 2, the caption incorrectly states that the R^2^ value is 0.0003 (sentence 6); however, this should state that the R^2^ value is 0.003.

In Figure 3, we mistakenly reported the slope as −0.4770 in the figure caption, whereas the correct value, consistent with what appears in the figure itself, is −0.07169. The figure caption also states an R^2^ range of 0.0006 to 0.085, when it should state 0.006 to 0.07. However, upon careful revision and in light of updated data, the original sentence was removed, as the information is no longer pertinent to the revised manuscript. Additionally, for Figure 3c (the adult subgroup), we initially stated that the regression was not statistically significant. However, upon rechecking, the regression is in fact statistically significant (*p* = 0.005), and the slope is steeper than originally noted, making the lung function decline in the adult subgroup greater than that in the pediatric subgroup. Nevertheless, this does not affect the discussion or conclusion of this article.

The corrected captions are listed below.

**Figure 1.** Baseline FEV_1_ percent predicted stratified by age group and sex in patients with PCD. (**a**) Adult patients exhibited significantly lower FEV_1_ values compared to pediatric patients, with a median of 48% [41–61%] predicted versus 71% [55–87%] predicted, respectively (*p* = 0.01). (**b**) Males had a higher median FEV_1_ of 65% [47–73%] predicted compared to females, whose median was 53% [43–71%] predicted (*p* = 0.50). Boxes represent the IQR, horizontal lines indicate medians, and whiskers extend to 1.5 times the IQR. Comparisons were performed using the Mann–Whitney U test. The asterisk (*) indicates statistical significance (*p* < 0.05); “ns” indicates not statistically significant.

**Figure 2.** Longitudinal change in FEV_1_ percentage predicted over time in patients with *RSPH4A*-associated primary ciliary dyskinesia. Scatterplot with individual patient trajectories and linear regression lines illustrating the relationship between FEV_1_ (% predicted) and months since diagnosis in patients with PCD. Each gray line represents repeated FEV_1_ measurements over time for a single patient. The black regression line reflects the overall trend, showing a slight, non-significant decline in FEV_1_ over time (slope = −0.06267). The shaded area represents the 95% confidence interval of the regression. The R^2^ value of 0.003 and *p*-value of 0.40 indicate no statistically significant association between FEV_1_ and time since diagnosis.

**Figure 3.** Stratified longitudinal trends in FEV_1_ among patients with *RSPH4A*-associated primary ciliary dyskinesia. Longitudinal changes in FEV_1_ (% predicted) are shown for patients stratified by sex and age group. Panel (**a**) displays data for male patients, (**b**) for female patients, (**c**) for adults (≥21 years), and (**d**) for pediatric patients (<21 years). Each gray line represents individual patient trajectories over time since diagnosis. The bold black line indicates the linear regression trend for each subgroup, with shaded areas denoting the 95% confidence interval. Only the regression model for adults demonstrated a significant association between FEV_1_ and time since diagnosis (*p =* 0.005).

Error in Figure/Table

In the original publication, there was a mistake in Table 1 as published. The interquartile range for “Age at diagnosis (years)” states [13–33.5], when it should state [13–39.5].

In Figure 2, the graph incorrectly states an “R^2^: 0.0003”, when it should be “R^2^: 0.003”.

Below are the corrected Table 1 and Figure 2.

**Figure 2:**
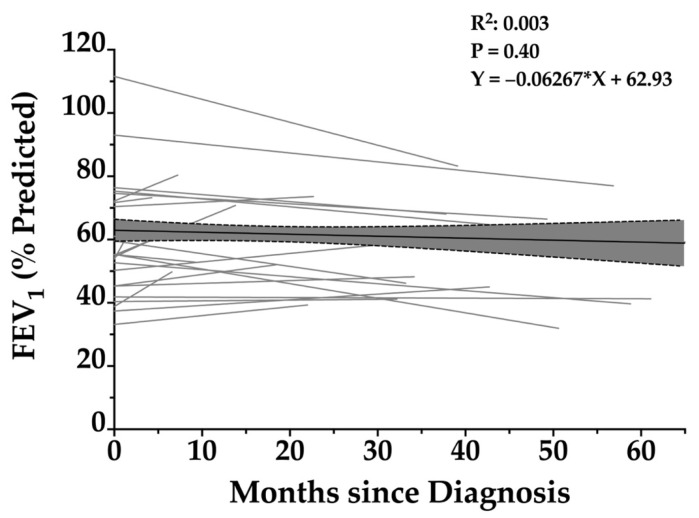
Longitudinal change in FEV_1_ percentage predicted over time in patients with *RSPH4A*-associated primary ciliary dyskinesia. Scatterplot with individual patient trajectories and linear regression lines illustrating the relationship between FEV_1_ (% predicted) and months since diagnosis in patients with PCD. Each gray line represents repeated FEV_1_ measurements over time for a single patient. The black regression line reflects the overall trend, showing a slight, non-significant decline in FEV_1_ over time (slope = −0.06267). The shaded area represents the 95% confidence interval of the regression. The R^2^ value of 0.003 and *p*-value of 0.40 indicate no statistically significant association between FEV_1_ and time since diagnosis.

Text Correction

There was an error in the original publication. In Section 3.2. Baseline Lung Function, first sentence of the second paragraph, we accidentally reported a *p*-value of 0.001, when it should be *p* = 0.01.

In Section 3.3. Longitudinal FEV_1_ Decline, sentence three of the second paragraph, there are three changes that must be corrected. The second interquartile bracket (paragraph 2, sentence 3) states [−0.34–0.16%], when it should say [−0.40–0.28%], and the adult patients median slope (paragraph 2, sentence 4) with its interquartile bracket, which states 0.14% [−0.01–1.13%], should state 0.16 [−0.06–1.14%]. In the fifth sentence of the second paragraph, the interquartile bracket says [−0.27–0.36%], when it should say [−0.27–0.75]. The corrected paragraph is presented below.

“To account for inter-individual variability, individual linear regressions were performed for each patient using their longitudinal spirometry data. The median of these patient-specific FEV_1_ slopes was 0.05% [−0.25–0.36%] predicted per year, with a wide range spanning from −0.73% to 5.04% per year, demonstrating significant heterogeneity in disease progression. When stratified by age group, pediatric patients (*n* = 11) exhibited a median FEV_1_ slope of −0.22% [−0.40–0.28%] per year, while adult patients (*n* = 14) had a median slope of 0.16% [−0.06–1.14%] per year. The difference between the median slope of adults and pediatrics was not statistically significant (*p* = 0.12). Stratification by sex showed that males had a median slope of 0.10% [−0.23–0.30%] per year, whereas females had a median slope of 0.02% [−0.27–0.75%] per year. No statistically significant difference in annual FEV_1_ decline between males and females was reached. Figure 3 shows the linear regressions of all stratifications”.

The authors emphasize that the scientific discussion and conclusions remain unchanged. This correction was reviewed and approved by the Academic Editor, and the original publication has been updated accordingly.

## Figures and Tables

**Table 1:** Demographic and clinical characteristics of the study cohort.

Characteristic	Value
Age at diagnosis (years)	20 [13–39.5]
Months since diagnosis	33.5 [11.7–46.2]
*RSPH4A* [c.921+3_6delAAGT]	25 (100%)
Bronchiectasis	25 (100%)
Pediatrics	11 (44%)
Adults	14 (56%)
Male	8 (32%)
Female	17 (68%)
